# Spindle cell renal cell carcinoma diagnosed after sunitinib treatment for chromophobe renal cell carcinoma

**DOI:** 10.1002/iju5.12135

**Published:** 2020-01-10

**Authors:** Ruriko Honda‐Takinami, Kei Ishibashi, Akifumi Onagi, Ryo Tanji, Kanako Matsuoka, Seiji Hoshi, Tomoyuki Koguchi, Junya Hata, Michihiro Yabe, Yuichi Sato, Hidenori Akaihata, Masao Kataoka, Soichiro Ogawa, Nobuhiro Haga, Yoshiyuki Kojima

**Affiliations:** ^1^ Department of Urology Fukushima Medical University School of Medicine Fukushima Japan

**Keywords:** chromophobe renal cell carcinoma, early tumor shrinkage, EMT, renal cell carcinoma, TKI

## Abstract

**Introduction:**

Chromophobe renal cell carcinoma presents in early pathological stages with a lower risk of metastasis. However, aggressive features and metastasis can occur. A rare case of rapidly progressive disease with histological changes is presented.

**Case presentation:**

A 56‐year‐old woman had a right renal tumor with multiple lymph node metastases, and the pathological diagnosis of the biopsy specimens from the primary tumor was chromophobe renal cell carcinoma. After sunitinib treatment, the metastatic lymph node had decreased in size and the numbers of circulating tumor cells were decreased, consequently, cytoreductive nephrectomy was performed. However, rapid progression of lymph node metastases was observed. Histopathological examination showed that the renal tumor was diagnosed as spindle cell renal carcinoma.

**Conclusion:**

It appears that the primary tumor underwent epithelial‐mesenchymal transition; further tissue specimen collection and analysis might be needed.

**Abbreviations & Acronyms**
CAM: cell adhesion molecule
CK‐FITC: cytokeratin fluorescein isothiocyanate
CT: computed tomography
CTC: circulating tumor cell
EMT: epithelial‐mesenchymal transition
EpCAM‐PE: epithelial cell adhesion molecule‐phycoerythrin
RCC: renal cell carcinoma

**Keynote message**

A case of transition from chromophobe RCC to spindle cell RCC after sunitinib treatment is reported. An association between a positive reaction to CD44 antibody on immunostaining of the primary tumor and the EMT was suggested.

## Case presentation

**Figure 1:**
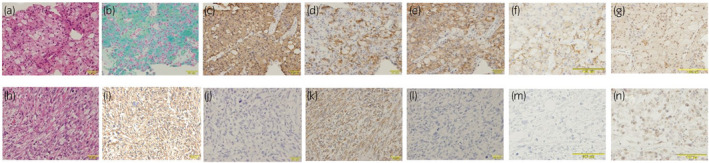
Immunohistochemical staining of biopsy specimens (a–g) and primary tumor after sunitinib treatment (h–n); hematoxylin and eosin stain (a,f), colloidal iron stain (b), CAM5.2 (c,h), vimentin (d,i), cytokeratin 7 (e,j), CD44 (g), E‐cadherin (f,m), N‐cadherin (g,n). Immunohistochemical staining of the biopsy shows positive reactions to colloidal iron stain, CAM5.2, and cytokeratin 7, and partially positive reaction to vimentin. After sunitinib treatment, immunohistochemical staining shows positive reactions to CD44, vimentin and N‐cadherin, but not cytokeratin 7, CAM5.2 and E‐cadherin.

A 56‐year‐old woman visited a hospital with a chief complaint of right abdominal pain on May 2015. Contrast enhanced CT showed a 7.8 × 4.6 cm right renal tumor, positron emission tomography‐magnetic resonance imaging also revealed multiple lymph node metastases but bone scintigraphy demonstrated no evidence of metastasis. She was diagnosed with T1bN2M0 renal tumor and referred to our department. Laboratory test of the serum was normal. Multicore biopsy of the primary tumor revealed that the pathological diagnosis of all of biopsy specimens was chromophobe RCC. Immunohistochemical staining showed positive reactions to colloidal iron stain, cytokeratin 7, CAM5.2, and vimentin (Fig. 1). Sunitinib was given as treatment for the advanced non‐clear cell RCC.

**Figure 2:**
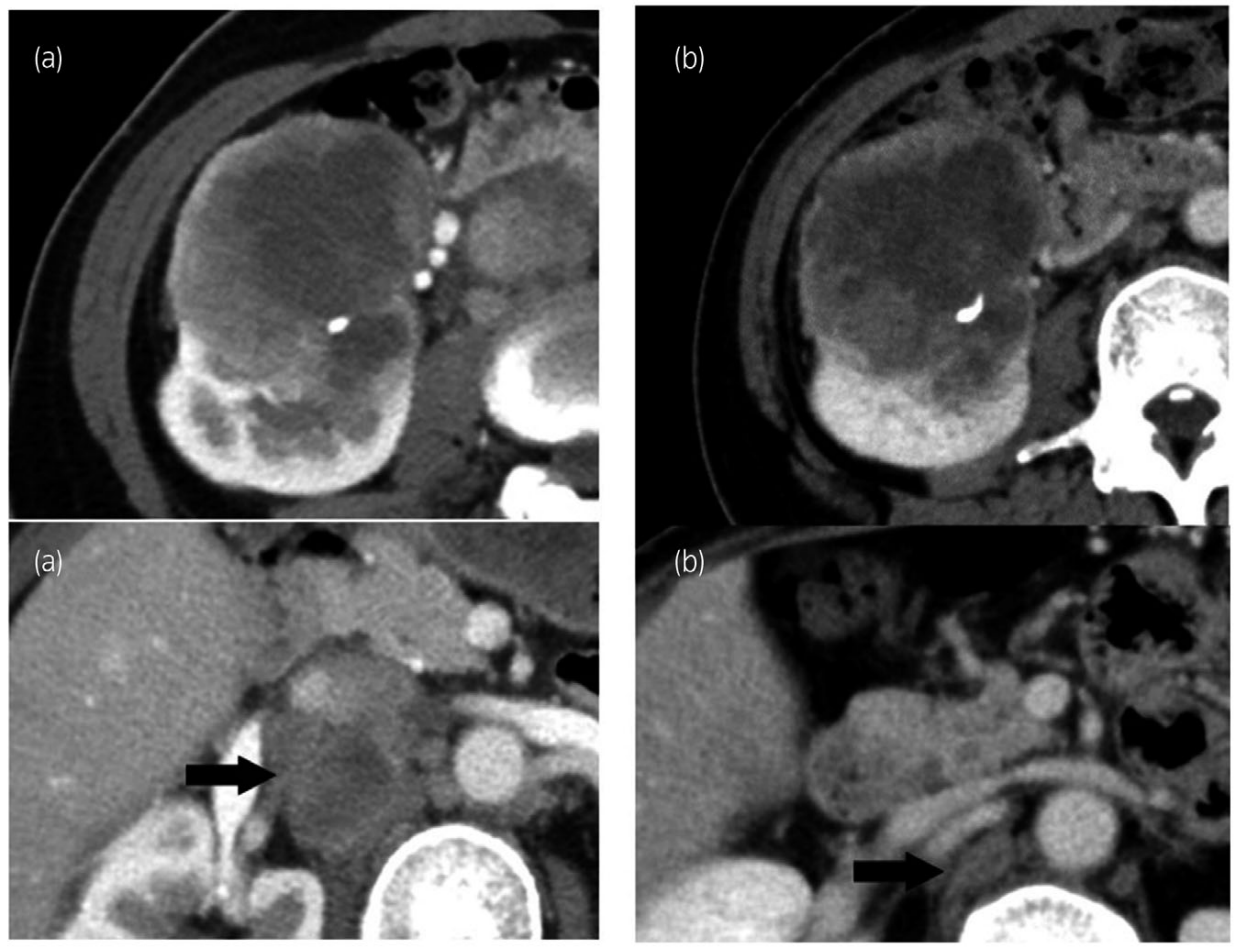
CT of the primary tumor and hilar lymph nodes before (a) and after (b) sunitinib treatment. Arrows indicate metastases. The metastatic lymph nodes have shrunk significantly.

After a total of 6 months of sunitinib treatment, CT showed that the metastatic lymph node had significantly reduced. The anatomical structure of renal hilar vessels was clarified by the sunitinib treatment (Fig. 2). Cytoreductive nephrectomy was performed. However, rapid progression of lymph node metastases was then observed, and axitinib was started as a second‐line therapy, because the sunitinib treatment caused a grade 3 adverse event of hand‐foot syndrome. No effect was observed with axitinib treatment, the disease progressed rapidly, distant lymph node metastases were seen, and the patient died 4 months after the operation. Histopathological examination showed spindle cell renal carcinoma with a necrotic region in the primary tumor, without a chromophobe RCC element in the primary tumor. Immunohistochemical staining showed a positive reaction to CD44, vimentin and N‐cadherin, but not cytokeratin 7, CAM5.2 or E‐cadherin (Fig. 1).

### Detection of CTCs

**Figure 3:**
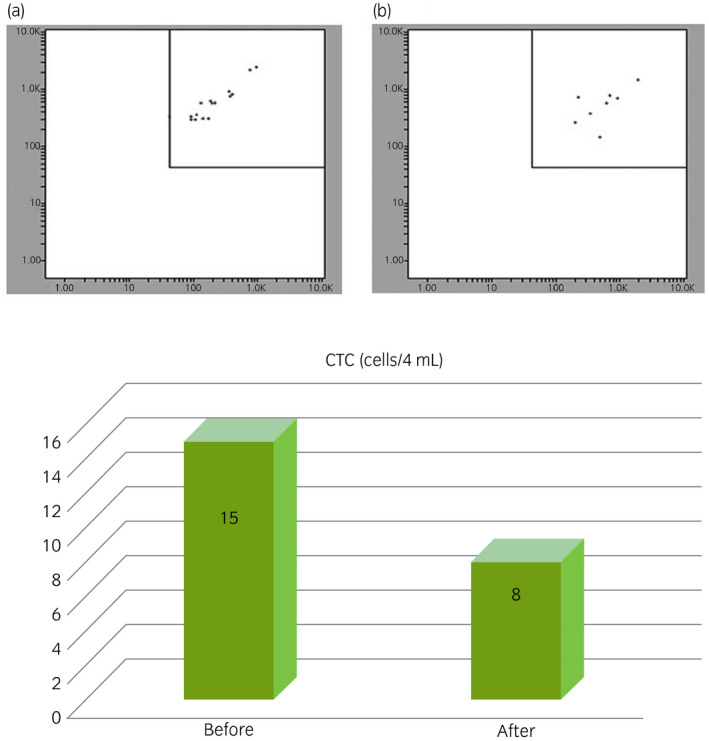
CTC analysis before (a) and after (b) sunitinib treatment. The number of CTCs before sunitinib treatment is 15 cells per 4 mL blood sample, and the number decreased to eight cells per 4 mL after 1 month.

The presence and number of CTCs were evaluated before and 1 month after sunitinib treatment to assess the efficacy of sunitinib, as described elsewhere.^1^ Briefly, blood samples were obtained from the patient and red blood cells were removed using Pharm Lyse^™^ lysing solution (On‐chip Biotechnologies, Tokyo, Japan). After negative selection with CD45‐conjugated microbeads, the cells were dissolved in a staining solution containing CK‐FITC, EpCAM‐PE. Flow cytometry was performed using On‐chip Sort (On‐chip Biotechnologies). In this case, the number of CTCs before sunitinib treatment was 15 cells per 4 mL blood sample, and the number decreased to eight cells 1 month after sunitinib treatment (Fig. 3). Consequently, the treatment was continued for five more months.

## Discussion

Chromophobe RCC is a rare tumor type accounting for approximately 5% of RCC cases.^2^ Chromophobe RCC often presents in early pathological stages with better nuclear grades and lower risk of metastasis, with 10‐year cancer‐specific survival >90% for localized disease.^3^ However, aggressive features and metastases can occur.^4^

The patient had received sunitinib as a first‐line treatment for advanced chromophobe RCC. A recent systematic review and meta‐analysis showed that sunitinib treatment for non‐clear RCC might be more effective than everolimus, but it also mentioned that the statistics supporting this statement were not yet entirely reliable.^5^ Although the effectiveness of sunitinib for non‐clear RCC has not yet been clearly established, a reduced number of CTCs and obvious lymph node shrinkage were observed in the patient with sunitinib treatment. Moreover, the sunitinib treatment enabled cytoreductive nephrectomy. However, the disease progressed rapidly and the subsequent axitinib therapy was not effective. It is of interest that only spindle cell renal carcinoma was found in the primary tumor, rather than chromophobe RCC that had been diagnosed by renal biopsy of the primary tumor before sunitinib treatment. Spindle cell renal carcinoma is also known as sarcomatoid RCC and shows aggressive behavior. It is understandable that the case that was diagnosed with spindle cell renal carcinoma, not chromophobe RCC, before sunitinib treatment, progressed rapidly because of its histological characteristics. However, the histological discrepancy between before and after sunitinib treatment should be mentioned. A plausible explanation for this finding is that the primary tumor underwent the EMT. It is reported that chromophobe RCC can develop a sarcomatoid transformation.^6^, ^7^ In advanced RCC, the transition of epithelial cells to sarcomatoid cells is considered to be the EMT in RCC.^8^ The EMT is characterized by loss of epithelial phenotype and decreased expression of E‐cadherin, a change of cell morphology into spindle‐like form and acquired motility capacity due to decreased expression of cytokeratins.^9^ In the present case, which showed positive reactions to CAM5.2 and cytokeratins, the reactions to those molecules turned negative after sunitinib treatment. In addition, decreased expression of E‐cadherin and high expression of N‐cadherin were observed in the primary tumor specimens that might indicate EMT in the tumor, although lack of positive staining in some chromophobe RCC was reported^10^ which was observed in our case. Recently progressed technologies to detect CTCs in RCC have received great attention based on their potential in evaluating the status of localized and metastatic diseases.^11^ It was previously reported that the presence of CTCs reflected the state of hematogenous metastasis.^12^ The report also mentioned that sunitinib treatment against metastatic RCC tumor could reduce the numbers of CTCs that might show that CTCs are expected to be used for a wide range of applications such as monitoring of therapeutic effects and early detection of drug resistance.^12^ In the present case, a reduced number of CTCs might not reflect the anti‐tumor effect of sunitinib treatment, because tumor cells that showed the EMT would not be captured by EpCAM and cytokeratin antibodies. Several studies have suggested the link between the EMT and a cancer stem‐like cell phenotype. CD44 is a marker of cancer stem‐like cells and is upregulated by chemotherapy in solid tumor cells.^13^ A drug‐induced cancer stem‐like cells phenotype might play a crucial role in the mechanism of acquisition of drug resistance.^14^ In this case, a positive reaction to CD44 antibody on immunostaining of the primary tumor, which was negative before sunitinib treatment, would indicate the EMT of the tumor.

Another possibility to explain this finding is that limited core biopsy before treatment might not have detected the spindle cell element. In this case, the chromophobe RCC element was considered to have disappeared with sunitinib treatment. Although it was limited to four core biopsies, why the prior biopsy could not detect the spindle cell element that occupied the whole primary tumor is puzzling. It is possible that rapid progression of the spindle cell element ensued after sunitinib treatment. Although further development might be needed, it would be interesting to analyze CTCs to detect EMT cells^1^ in RCC treatment.

In conclusion, this was a case of spindle cell RCC which would indicate sarcomatoid transformation after sunitinib treatment. Immunohistochemical staining for CD44 indicated the EMT in the chromophobe RCC. Further tissue specimen collection before and after sunitinib treatment might be needed to clarify this further.

## Conflict of interest

The authors declare no conflict of interest.
